# Application of Mootral^TM^ Reduces Methane Production by Altering the Archaea Community in the Rumen Simulation Technique

**DOI:** 10.3389/fmicb.2018.02094

**Published:** 2018-09-04

**Authors:** Melanie Eger, Michael Graz, Susanne Riede, Gerhard Breves

**Affiliations:** ^1^Institute for Physiology, University of Veterinary Medicine Hanover, Hanover, Germany; ^2^Neem Biotech Ltd., Abertillery, United Kingdom

**Keywords:** Illumina, methanogens, RUSITEC, garlic, citrus, organosulphur, polyphenols

## Abstract

The reduction of methane emissions by ruminants is a highly desirable goal to mitigate greenhouse gas emissions. Various feed additives have already been tested for their ability to decrease methane production; however, practical use is often limited due to negative effects on rumen fermentation or high costs. Organosulphur compounds from garlic (*Allium sativum*) and flavonoids have been identified as promising plant-derived compounds which are able to reduce methane production. Here, we evaluated the effects of a combination of garlic powder and bitter orange (*Citrus aurantium*) extracts, Mootral, on ruminal methane production, ruminal fermentation and the community of methanogenic *Archaea* by using the rumen simulation technique as *ex vivo* model. The experiment consisted of an equilibration period of 7 days, an experimental period of 8 days and a withdrawal period of 4 days. During the experimental period three fermenters each were either treated as controls (CON), received a low dose of Mootral (LD), a high dose of Mootral (HD), or monensin (MON) as positive control. Application of Mootral strongly reduced the proportion of methane in the fermentation gas and the production rate of methane. Moreover, the experimental mixture induced a dose-dependent increase in the production rate of short chain fatty acids and in the molar proportion of butyrate. Some effects persisted during the withdrawal period. Both, single strand conformation polymorphism and Illumina MiSeq 16S rRNA amplicon sequencing indicated an archaeal community distinct from CON and MON samples in the LD and HD samples. Among archaeal families the percentage of *Methanobacteriaceae* was reduced during application of both doses of Mootral. Moreover, several significant differences were observed on OTU level among treatment groups and after withdrawal of the additives for LD and HD group. At day 14, 4 OTUs were positively correlated with methane production. In conclusion this mixture of garlic and citrus compounds appears to effectively reduce methane production by alteration of the archaeal community without exhibiting negative side effects on rumen fermentation.

## Introduction

Livestock emissions are responsible for about 15% of all global greenhouse gas emissions and are the third largest contributor of greenhouse gasses after energy and industry (Gerber et al., 2013). Beef and dairy cattle are the major contributors to enteric methane production from livestock species (EPA, 2017). As the production of methane in the rumen can represent a loss of up to 12% of the digestible energy (Johnson and Johnson, 1995), it is of great interest to reduce methane production in cattle or ruminant husbandry in general. Research on feed supplementation is currently being intensively pursued with a view to improving energy utilization in ruminants and mitigating the production of methane by these animals.

Ruminal methane production is performed by methanogens, a sub group of the *Archaea* which are characterized by their ability to produce methane as the end product of the metabolism of hydrogen, carbon dioxide or formate released during bacterial degradation of the feed. The removal of hydrogen from the rumen during methanogenesis facilitates the optimum performance of the bacteria involved in the fermentation as it supports the complete oxidation of substrates which would not be possible if the hydrogen was not removed (reviewed by Bodas et al., 2012). Moreover, there are some methanogens that can use methanol and methylamines to produce methane (Patterson and Hespell, 1979).

Manipulation of the rumen microbiome using dietary interventions, e.g., modulation of the diet composition or application of natural or chemical feed additives, to reduce methane production and increase productivity has been shown to be effective (Denman et al., 2007; Lettat et al., 2013; Belanche et al., 2016), although has yet not been implemented significantly in the agricultural sector due to certain limitations like microbial adaptation, practicability, residues, costs or negative effects on feed intake, feed degradability and productivity (Kumar et al., 2014). Dietary interventions using natural products have long attracted attention as potential agents in the mitigation of methane production (Chaves et al., 2008; Khiaosa-Ard et al., 2015; Belanche et al., 2016) and have been demonstrated to be attractive alternatives to synthetic chemicals in animal health.

Biological responses to the organosulphur compounds from garlic include well documented antimicrobial properties (Ankri and Mirelman, 1999) and hence garlic extracts and several of its compounds have been researched extensively to modify the rumen microbiome, but to date have been reported to exhibit variable methane reducing capacity in ruminants in *in vitro* and *in vivo* studies (Busquet et al., 2005b; Chaves et al., 2008; Patra et al., 2011; Ma et al., 2016). Soliva et al. (2011) demonstrate a nearly complete inhibition of methanogenesis using a garlic oil distillate without impacting OM degradation in a Rusitec experiment. Busquet et al. (2005a) postulated that garlic extracts affect ruminal *Archaea* directly, and this hypothesis is supported by subsequent studies, e.g., observing effects of garlic oil on the diversity of methanogenic archaea in sheep rumen (Ohene-Adjei et al., 2008). However, the stability of organosulphur compounds from garlic in liquid formulations is limited (Yoo et al., 2014) and impairs its practical usage.

Another group of plant derived compounds which have potential for dietary use to reduce methane emissions are flavonoids. Flavonoids are a large group of polyphenolic compounds which have been long used in human medicine for their anti-inflammatory, antioxidant and antimicrobial properties (reviewed by Kumar and Pandey, 2013). Dietary addition of plant extracts high in flavonoids and other polyphenols, e.g., from citrus fruits, may reduce susceptibility of cattle to rumen acidosis by promoting certain rumen bacteria and thereby stabilizing ruminal fermentation (Balcells et al., 2012). Moreover, flavonoids suppress methane production and exhibit a direct effect on rumen methanogens and protozoa (Oskoueian et al., 2013). The antioxidative activity of flavonoid containing plant extracts supports the prevention of lipid peroxidation in lactating dairy cattle (Gobert et al., 2009).

Considering the impacts of garlic extracts and flavonoids on reducing rumen methanogenesis, we postulated that combining the organosulphur compounds from garlic with the polyphenols contained in citrus, would lead to a persistent change in the rumen archaeal microbiome resulting in the reduction of methanogenesis. To overcome the limitations of garlic oil we used a commercial mixture of dried garlic and citrus extract. We analyzed the effects of this mixture on methane production, ruminal fermentation and the archaeal community using the Rusitec. Monensin was used as a positive control, as it is known to alter rumen methanogenesis by selecting for Gram-negative bacteria in the rumen resulting in a reduction in methanogenesis and an increase in propionate production (Russell and Strobel, 1989).

## Materials and Methods

### Ethics Statement

All procedures involving animals were carried out in accordance with the German Legislation for Animal Welfare. The fistulation of donor cows was approved in 2013 according to the regulations in force at the time by the Lower Saxony State Office for Consumer Protection and Food Safety (33.42502-05-07A480).

### Plant Material

The garlic (*Allium sativum*) components for the experiments were developed from commercial powder (SinoBNP, Qingdao, China). The powder was standardized to contain 1.5% (w/w) Allicin (S-Prop-2-en-1-yl prop-2-ene-1-sulfinothioate). Allicin concentrations were analyzed by HPLC (Agilent Technologies 1100 Series, Wokingham, United Kingdom) using a C18 (250 mm × 4.6 mm, 5 μm) column with a flow rate of 1 mL/min with the mobile phase being 50:50 HPLC grade methanol and water and UV detection at 210 nm. The sample diluent was HPLC water. The citrus components for the experiments were developed from commercially available extracts (Khush Ingredients, Oxford, United Kingdom) of bitter orange (*Citrus aurantium*). The total polyphenol content of the citrus extract was standardized to 45% (w/w) by the Folin-Ciocalteau Method (Kaur and Kapoor, 2002). Flavonoid concentrations were analyzed by HPLC (Agilent Technologies 1100 Series, Wokingham, United Kingdom) using a C18 (250 mm × 4.6 mm, 5 μm) column with a flow rate of 1 mL/min with the mobile phase being a gradient of HPLC grade methanol and water and UV detection at 340 nm. The sample diluent was 80% DMSO (Sigma-Aldrich Ltd., Dorset, United Kingdom). Standards used were Naringin, Naringenin, Neohesperidin, Rhoifolin, and Neoeriocitrin (Sigma-Aldrich Ltd., Dorset, United Kingdom). For Mootral, the material was premixed at a ratio of nine parts garlic powder to one part citrus powder and subsequently added to the feed material.

### Rumen Simulation Technique (Rusitec) Experiment

The experiment was divided into three distinct phases: an equilibration phase of 7 days to allow the Rusitec system to stabilize, an EP of 7 days and a WP of 4 days to consider any long term effects of the rumen modifiers being investigated. Twelve fermentation vessels were used simultaneously. Three vessels each were designated as controls (CON, vessels A, F, L), received a 1 g dose of the experimental mixture (LD, vessels B, G, K), a 2 g dose of experimental mixture (HD, vessels C, E, I) or a dose of 4.1 mg Monensin (MON, vessels D, H, J). All treatments were administered once daily in the experimental period together with the feed material. In the WP, no further additions of modifiers were made.

For the start of the Rusitec experiment, rumen contents were collected in the morning (about 3 h after feeding) from two ruminal fistulated German Holstein Cows (age: 6 years). Solid content was separated from the liquid by gauze-filtration. Rumen contents from both cows were mixed and maintained at 39°C throughout the preparation. The Rusitec experiment was carried out as described by Czerkawski and Breckenridge (1977). The daily substrate consisted of 7 g hay, cut into pieces of 1.5–2 cm in length, and 3 g concentrate (Deuka Schaffutter, Deutsche Tiernahrung Cremer, Düsseldorf). Crude nutrients of the feed are presented in **Supplementary Table S1**. Starting the experiment, one nylon bag (11.5 cm × 6.5 cm, pore size 150 μm) was filled with 70 g of the solid phase of the rumen content and a second nylon bag with the substrate. Both nylon bags were placed into the inner vessel of the fermentation vessel, the liquid phase of the rumen content was poured into the outer vessel. The inner vessel was continuously moved up and down by an electric motor (6 times/min). After 24 h, the nylon bag containing the solid phase was replaced by a substrate-filled one. The nylon bags were replaced alternately with a new substrate bag every 24 h, such that the retention time for each nylon bag was 48 h. The fermentation vessel was continuously infused with a buffer solution (**Supplementary Table S2**) to achieve a liquid turnover of once per day (dilution rate 4.2%/h). The effluent was collected in conical flasks kept on ice and the fermentation gas was collected in gas bags (Plastigas, Linde AG, Munich, Germany). After the daily exchange of the substrate bag and sampling, the collections flasks were flushed with nitrogen to maintain anaerobic conditions.

### Sampling and Sample Analysis

Adequate environmental conditions for microbial survival and the anaerobic status of the system were ensured by daily measurements of pH values and redox potentials during the whole experiment. During EP and WP effluent samples were collected daily for the analysis of NH_3_-N and SCFA concentrations and stored at −20°C until further analysis. NH_3_-N concentrations were determined photometrically at 546 nm in a spectrometer (DU 640, Beckman Coulter GmbH, Krefeld, Germany) as described by Riede et al. (2013). Analysis of SCFA concentrations was performed by gas chromatography as described previously (Koch et al., 2006). Production rates of SCFA were calculated by multiplying SCFA concentration with daily effluent volume. Substrate residues were collected and dried at 65°C for 48 h. In order to determine the degradation of OM, dry matter was incinerated in a muffle furnace at 600°C for 24 h. Gas volume was determined using a drum type meter (Ritter Apparatebau, Bochum, Germany). Gas samples were collected in glass collection tubes and analyzed for percentages of CH_4_ and CO_2_ with a gas chromatograph (GC 2014, Shimadzu Europa GmbH, Duisburg, Germany) at the Institute of Sanitary Engineering and Waste Management, University of Hanover (Hanover, Germany) using a thermal conductivity detector, a glass column filled with Porapak Q (Sigma-Aldrich GmbH) and He as carrier gas. Free hydrogen was not measured. CO_2_ and CH_4_ production was calculated by multiplying CO_2_ and CH_4_ percentages with total gas volume corrected for standard conditions (0°C; 1013 hPa). Hydrogen production was calculated using the formula by Wang et al. (2014) derived from the equations from Demeyer (1991):

$$\begin{array}{l} {\text{NetH}}_{2}(\text{mM})=2(\text{acetate}+\text{n-butyrate}+\text{iso}-\text{butyrat}\\ \;-\text{propionate}+\text{iso}-\text{valerate}+\text{valerate} \end{array}$$

### DNA Isolation

On days 14 and 18, respectively, 30 ml of liquid was removed from each of the 12 fermentation vessels. Samples were centrifuged once at 2000 × *g*, 5 min, 4°C to remove feed particles. The effluent was then centrifuged at 27000 × g, 20 min, 4°C, and the effluent was discarded while the remaining pellets was washed three times using sterile 0.9% NaCl solution (27000 × *g*, 20 min, 4°C). The samples were frozen in liquid nitrogen and stored at −80°C until DNA isolation. DNA isolation was carried out as described by Meibaum et al. (2012), with the extraction of genomic DNA optimized with an additional purification step using the peqGold Tissue DNA Kit (VWR International GmbH, Darmstadt, Germany). Quantity and purity of genomic DNA was measured using the Nanodrop One Spectrophotometer.

### Single Strand Conformation Polymorphism

The SSCP analysis was used for detecting effects of the experimental mixture and Monensin on the population of *Archaea*. The SSCP procedure followed the protocol by Meibaum et al. (2012). Briefly, the first PCR was used for amplification of 16S rRNA gene fragments of *Archaea* by using the primer pair A109f [AC(G/T) GCT CAG TAA CAC GT] (Großkopf et al., 1998) and A934b reverse (GTG CTC CCC CGC CAA TTC CT) (Stahl and Amann, 1991). The total reaction volume was 25 μl with a final concentration of 1x PCR buffer with 2.5 U/μl HotStar HiFidelity DNA polymerase (Qiagen, Hilden, Germany) and 25 ng template DNA. The PCR consisted of an initial denaturation of 15 min at 95°C followed by 30 cycles of denaturation at 94°C for 60 s, annealing at 52°C for 60 s and elongation at 72°C for 70 s. The final elongation was performed at 72°C for 5 min. For the nested PCR, the primers Com1m forward [CAG C(A/C)G CCG CGG TAA (C/T)AC] and Com 2m-Ph reverse (CCG CCA ATT CCT TTA AGT TT) (Boguhn et al., 2010) were used. Reverse primers were phosphorylated at 5′ end for further single strand digestion. The second PCR was carried out with the following conditions: initial denaturation 15 min at 95°C, 25 cycles of denaturation at 94°C for 45 s, annealing at 56°C for 45 s, elongation at 72°C for 60 s, final elongation at 72°C for 5 min. Gel electrophoresis of single strand DNA was carried out at 20°C and 300 V for 22.5 h. Polyacrylamide (0.625%) SSCP gels were air dried and scanned (ScanMaker i800; Mikrotek, Willich, Germany).

### Statistical Analysis

Statistical analysis of biochemical parameters was performed using Graph Pad Prism 7.02 (Graph Pad Software, San Diego, CA, United States). For statistical analysis, the last day of EP (day 14) and CP (day 18) were compared. Due to missing values day 17 was used instead of day 18 for the degradation of OM. Data were assessed for normal distribution of residuals by applying the Kolmogorov–Smirnov test. Two-way ANOVA for repeated measurements was applied to detect effects of Time, Treatment, or interactions of Time × Treatment. In case of significant interaction, Sidak post-test was used to identify significant differences among treatments within time-points and between time-points within treatment. In case of significant effects of treatment without interaction, Sidak post-test was applied to detect differences among treatments for both time-points. Significance levels were set at *P* < 0.05 (^∗^), *P* < 0.01 (^∗∗^), *P* < 0.001 (^∗∗∗^). Tendencies were defined as *P* < 0.1 (*t*).

Results of the SSCP analysis are depicted as NMDS-plots and cluster analysis with dendrograms based on similarity matrices calculated using the Pearson product-moment correlation coefficient. The clustering algorithm UPGMA (Unweighted Pair Group Method Using Arithmetic Mean) was used. A non-parametric multivariate analysis of variance suitable for statistical analysis of non-normal and discontinuous data was carried out using dissimilarity matrices as described previously (Anderson, 2001). Calculations were conducted using the software PERMANOVA. Differences between treatments were regarded as significant at *P* < 0.05 (Anderson and Robinson, 2003).

### 16S DNA Illumina Sequencing and Data Analysis

For sequencing of the hypervariable regions V3/4 of the archaeal 16S rRNA gene, two-step PCR libraries were created using the primer pair Arch349F (5′- GYG CAS CAG KCG MGA AW-3′) and Arch806R (5′- GGA CTA CVS GGG TAT CTA AT-3′) (Takai and Horikoshi, 2000). The Illumina MiSeq platform and a v2 500 cycles kit were used to sequence the PCR amplicon libraries. The produced paired-end reads which passed Illumina’s chastity filter were subject to de-multiplexing and trimming of Illumina adaptor residuals using Illumina’s real time analysis software (no further refinement or selection). The quality of the reads was checked with the software FastQC version 0.11.5. The locus specific adaptors were trimmed from the sequencing reads with the software cutadapt v1.14. Paired-end reads were discarded if the adaptor could not be trimmed. Trimmed forward and reverse reads of the paired-end reads were merged using the software USEARCH version v10.0.240. Merged sequences were then quality filtered allowing a maximum of one expected error per merged read and by discarding those containing ambiguous bases. The remaining reads were grouped into OTUs by the “denoising” algorithm implemented in USEARCH (Edgar, 2016), discarding singletons and chimeras in the process. OTUs with an abundance of less than 0.001 of the filtered read amount of all samples were discarded. The remaining OTUs were then aligned against the reference sequences of the Silva v123 database and taxonomies were predicted considering a minimum confidence threshold of 0.7 by the USEARCH “sintax” algorithm. Alpha and beta diversity calculations and the rarefaction analysis were performed with the software phyloseq v1.16.2. Intra community diversity was estimated using the Richness (Observed), Chao1 and Shannon indices. Inter community diversity was estimated using the unifrac distance measure. Differential OTU analysis on normalized abundance counts contrasting the different experimental groups was performed with the software DESeq2 v1.12.4. Libraries, sequencing and data analysis were performed by Microsynth AG (Balgach, Switzerland). Intra community diversity and relative abundances were compared using One-way ANOVA and relationships between normalized abundance of archaeal OTUs and methane production were analyzed using Spearman correlation in Graph Pad Prism. For further taxonomic classification the 15 most abundant OTUs (relative abundance ≥ 1%) were additionally compared to the 16S sequence data base of the JGI Integrated Microbial Genomes and Microbiomes (IMG/M) portal^1^ using the integrated BLAST tool.

### Accession Number

Illumina MiSeq sequencing data are available in BioProject SRA database under the accession number PRJNA472698.

## Results

### Effect of the Experimental Mixture on Bacterial Fermentation

The experimental treatment affected several biochemical parameters of rumen fermentation (**Table 1**). The pH was slightly lower in LD and HD group compared to MON group particularly at day 14 (*P* < 0.01). However, neither the fermenters treated with the experimental compound, nor with Monensin differed in pH compared with the CON fermenters and all values were within the physiological range. Furthermore, the redox potential was more negative in HD14 compared to MON14 (*P* < 0.01). The concentration of NH_3_-N tended to be affected by Interaction of Time × Treatment with numerically highest values in HD14 samples. The total daily production rate of SCFAs was increased in HD group compared with CON group and MON group (at least *P* < 0.01) and was higher in LD group compared with the MON group throughout the experiment (*P* < 0.01), with slightly lower levels in the WP. The individual SCFA were affected by the treatment to differing extents resulting in alterations in the composition of the SCFA mixture. Both, LD and HD treatment had no impact on the molar proportion of acetate, however, HD treatment slightly decreased the percentage of propionate compared with CON (*P* < 0.05). Furthermore, the HD treatment resulted in an increase in butyrate proportion (*P* < 0.05 compared with CON) at day 14. The LD treatment led to an intermediate butyrate proportion in the EP. In contrast, MON clearly reduced the molar proportions of acetate and butyrate (at least *P* < 0.01 compared with all other groups) and significantly increased the proportion of propionate (*P* < 0.001 compared with all other groups). The proportion of isovalerate was elevated by HD treatment at day 14 (at least *P* < 0.01), while MON application increased the percentage of valerate throughout the experiment (at least *P* < 0.01). MON led to a reduction of the isovalerate concentration below the detection limit, therefore molar proportions were not determined. The net hydrogen production calculated based on the concentrations of the fatty acids was significantly enhanced for the HD group compared with CON (*P* < 0.01), while it was intermediate between CON and HD samples for LD. MON significantly reduces hydrogen production compared with CON, LD, and HD treatment (at least *P* < 0.01). Moreover, the increase in SCFA production was accompanied by an enhanced degradation of OM at day 14 in the HD group compared with the MON group (*P* < 0.001, **Table 1**).

**Table 1:** Fermentation parameters at day 14 (experimental period) and day 18 (/17 withdrawal period).

Parameter	day	Treatment				Pooled *SD*	*P*-value		
		CON	LD	HD	MON		Treatment	Time	Interaction
pH	14	6.79^AB^	6.66^A^	6.66^A^	6.87^B^	0.05	0.0016^2^	<0.001	0.072
	18	6.85^AB^	6.82^A^	6.79^A^	6.90^B^	0.04			
Redox potential	14	−264^Ab^	−267^Ab^	−300^A^	−244^B∗^	19.9	n.s.	0.036	0.0088
	18	−291	−280	−271	−288^∗^	12.8			
NH_3_-N [mM]	14	6.30	6.74	9.94	6.02	1.01	n.s.	n.s.	0.078
	18	7.80	7.41	6.54	6.69	1.56			
SCFA total [mmol/d]	14	21.21^AC^	30.86^AB^	36.31^B^	16.46^C^	4.10	<0.001	0.0042	n.s.
	18	18.24^AC^	20.71^AB^	24.50^B^	15.38^C^	3.39			
Acetate [%]	14	59.00^A^	56.66^A^	54.59^A^	51.49^B^	1.67	<0.001	0.0096	0.076
	18	58.34^A^	58.91^A^	58.46^A^	52.97^B^	1.01			
Propionate [%]	14	25.28^A^	24.90^AB^	22.54^B^	38.43^C^	1.24	<0.001	n.s.	n.s.
	18	25.64^A^	24.78^AB^	23.58^B^	37.20^C^	0.80			
Butyrate [%]	14	10.57^A^	12.24^Ab∗^	15.07^B∗^	4.04^C^	1.96	<0.001	0.0010	<0.001
	18	10.85^α^	10.78^α∗^	11.52^α∗^	4.32^β^	1.27			
Isovalerate [%]	14	2.37^A^	2.68^A^	4.25^B∗^	n.d.	0.53	0.029	0.0010	0.0082
	18	2.24	2.25	2.87^∗^	n.d.	0.38			
Valerate [%]	14	2.78^A^	3.51^A^	3.57^A^	6.04^B^	0.55	<0.001	n.s.	n.s.
	18	2.94^A^	3.28^A^	3.57^A^	5.51^B^	0.54			
Net H_2_ production [mM]^1^	14	36.55^A^	51.07^AB^	60.89^B^	16.43^C^	4.75	<0.001	0.031	0.074
	18	30.16^A^	34.41^AB^	42.41^B^	17.07^C^	6.64			
Degradation of organic matter [%]	14	41.96^Ab^	40.90^Ab^	50.44^A^	31.09^B∗^	5.63	0.0046	n.s.	0.022
	17	42.30	40.47	37.76	35.95^∗^	2.84			

*^1^Net H_2_ production was estimated by stoichiometric calculation using the formula by (Demeyer, 1991; Wang et al., 2014). ^2^Results of the statistical analysis of pH, redox potential and NH_3_-N have been included in a conference abstract previously (Eger et al., 2018). During the experimental period control fermenters (CON) received no addition, low dose group (LD) was treated with 1 g of the experimental mixture, high dose fermenters (HD) received 2 g of the experimental mixture and monensin was used as positive control (MON). During withdrawal period no additions were made. Significant differences (at least P < 0.05) are indicated by different capital letters among treatment groups for both time-points, by small letter among treatment groups at day 14 and by Greek letters among treatment groups at day 18. Asterisks indicate significant differences within treatment group between time-points. Data are presented as means and pooled SD. n.d. = not detectable.*

### Gas Production

**FIGURE 1:**
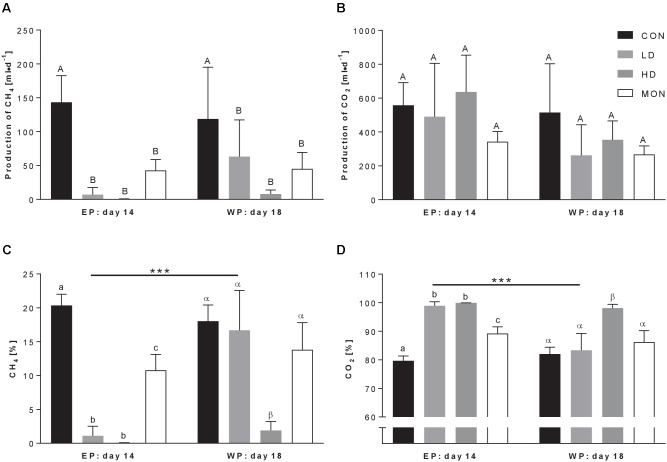
Production of fermentation gas at the last day of experimental period (EP) and withdrawal period (WP). The daily production rate of methane **(A)** and carbon dioxide **(B)** was calculated by multiplying the proportions of methane **(C)** and carbon dioxide **(D)** in the fermentation gas with the volume of fermentation gas corrected for standard conditions (1013 hPa, 0°C). Effects of a low dose (LD, light gray bars) and a high dose (HD, dark gray bars) of the experimental mixture were compared with a negative control (CON, black bars) and a positive control (monensin, MON, white bars). Two-way ANOVA followed by Sidak post-test was applied to detect significant effects of the factors Time, Treatment or interaction Time × Treatment. ANOVA revealed significant effects of Treatment (*P* = 0.001) on methane production and of Treatment (both *P* < 0.001), Time (both *P* = 0.003) and interaction of Time × Treatment (both *P* = 0.02) on the percentages of methane and carbon dioxide. Carbon dioxide production only tended to be affected by Time (*P* = 0.076). Significant differences in Sidak post-test are indicated using different capital letters, small letters and Greek letters for differences among treatment groups for both time-points, among treatment groups in experimental period and among treatment groups in withdrawal period (at least *p* < 0.05), respectively. Time-dependent alterations within groups are indicated by asterisks. ^∗∗∗^*P* < 0.001. Data are presented as means ± SD. Number of replicates *n* = 3.

The application of feed additives significantly affected methane production (Treatment *P* = 0.0011, **Figure 1A**). As expected, methane production was reduced in MON group compared to CON group (42.6 ± 16.1 ml/d vs. 143.3 ± 39.4 ml/d, mean ± SD). Furthermore, application of the experimental mixture decreased the production of methane significantly, regardless of the dose (*p* < 0.01, 6.9 ± 10.7 ml/d for LD, 0.43 ± 0.40 ml/d for HD). This effect persisted during the withdrawal period for all three treatments (Time *P* = 0.55, Interaction *P* = 0.44). In contrast, the production rate of carbon dioxide was not significantly affected by treatment (**Figure 1B**). The percentage of methane in fermentation gas was reduced in both Mootral treated groups (LD and HD) compared with CON group (*P* < 0.001) and compared with MON group (*P* < 0.01) (**Figure 1C**) indicating an even stronger inhibitory effect on methane production than by Monensin (CON vs. MON *P* < 0.01). This effect persisted during the WP for the HD treatment, however, not for the LD treatment (LD14 vs. LD18 *P* < 0.001). Due to the reduced methane production, the percentage of carbon dioxide in the fermentation gas mixture was increased in the vessels treated with feed additives (Treatment *P* < 0.001, **Figure 1D**).

### SSCP Analysis

**FIGURE 2:**
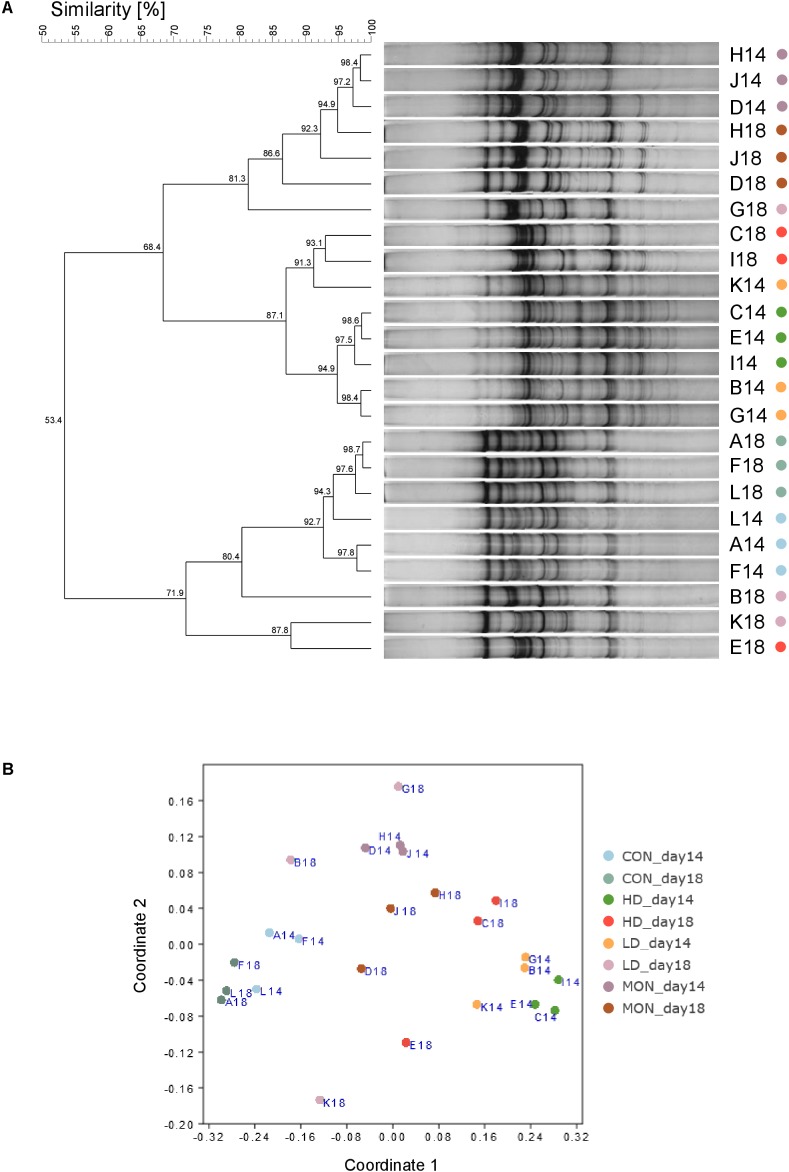
Similarities of archaeal communities analyzed by single strand conformation polymorphism. Dendrogram analysis based on UPGMA clustering was used to compare similarities of band patterns on the SSCP gel **(A)** and the similarity matrix was visualized as NMDS plot (**B**, Stress = 0.09). During the experimental period fermenters A, F, L received no addition (CON14, light blue), fermenters B,G,K received 1 g of the experimental mixture (LD14, orange), fermenters C, E, I were treated with 2 g of the experimental mixture (HD14, dark green) and 4.1 mg Monensin was added to fermenters D, J, H (MON14, dark purple). During withdrawal period no additions were made (CON18, blue green; LD18, light purple; HD18, red; MON18, brown).

Results of the SSCP analysis were depicted as NMDS plot and statistically analyzed by PERMANOVA. Significant differences were revealed for the factors Time (*P* < 0.01), Treatment (*P* < 0.001) and Interaction Time × Treatment (*P* < 0.01). At day 14, replicates of each treatment clustered close together (**Figure 2B**). In agreement with the statistical analysis, CON samples clustered separately from MON treated samples, LD and HD (each *P* < 0.001) samples in the NMDS plot. In addition, MON samples clustered separately from LD (*P* < 0.001) and HD (*P* < 0.001) samples, while fermenters with both doses of the experimental mixture clustered next to each other. A shift in the SSCP profile was observed between days 14 and 18 for CON group (*P* < 0.05), LD group (*P* < 0.05), and HD group (*P* < 0.05), while no differences were observed for MON group. Although a significant shift was detected in the CON group between both phases, these samples clustered relatively close together in the plot and exhibited a 92.7% similarity (**Figure 2A**), indicating that this result is probably based on the low variety in CON18 samples (97.6%) and without biological importance. In contrast, samples in the LD and HD group depicted an observable time-dependent shift in the NMDS plot and a greater variety within group at day 18. Moreover, HD and MON samples were still different from CON (*P* < 0.01) samples, while LD samples were no longer significantly different from the other groups.

### Illumina Amplicon Sequencing

#### Archaeal Community Composition

Sequencing resulted in a total of 558,522 reads after quality filtering. Two samples were removed from further analysis due to low read count (LD14: I14, HD14: K14). Read count ranged from 1,151 to 36,430 reads with a mean of 25,387. In total, 44 OTUs were identified of which 33 were classified within the domain *Archaea* (**Supplementary Table S3**) and 11 within *Bacteria* (**Supplementary Table S4**). Despite the detection of 11 bacterial OTUs, the overall abundance of *Archaea* in all samples was 96.4%. All archaeal OTUs belonged to the phylum *Euryarchaeota*. Within these OTUs, 14 could be assigned to the family level (class *Thermoplasmata*, order *Thermoplasmatales*, family *Thermoplasmatales Incertae Sedis*), 17 could be assigned to the genus level with 12 OTUs from genus *Methanobrevibacter* and 3 from genus *Methanosphaera* (class *Methanobacteria*, order *Methanobacteriales*, family *Methanobacteriaceae*) and 2 belonging to the genus *Methanomicrobium* (class *Methano microbia*, order *Methanomicrobiales*, family *Methanomicro biaceae*). Two archaeal OTUs could be assigned to the species level, one from genus *Methanobrevibacter* (methanogenic archeon LGM-SL1) and one from genus *Methanosphaera* (*Methanosphaera* sp. ISO3-F5).

#### Intra and Inter Community Diversity

**FIGURE 3:**
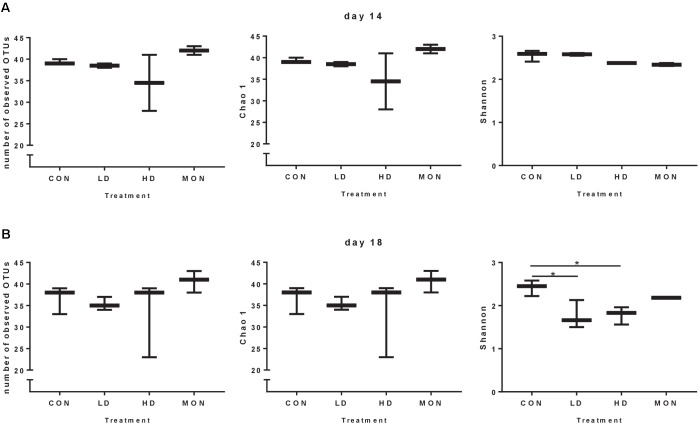
Number of observed operational taxonomic units (OTUs), richness and diversity of the archaeal community under different treatment conditions during the experimental period (day 14, **A**) and the withdrawal period (day 18, **B**). Data are shown as Box and Whiskers with Min to Max range. Results were analyzed by One-way ANOVA for day 18. A significant effect of the Treatment was observed for Shannon index at day 18 (*P* = 0.014). Asterisks indicate significant differences in Tukey post-test with *P* < 0.05.

**FIGURE 4:**
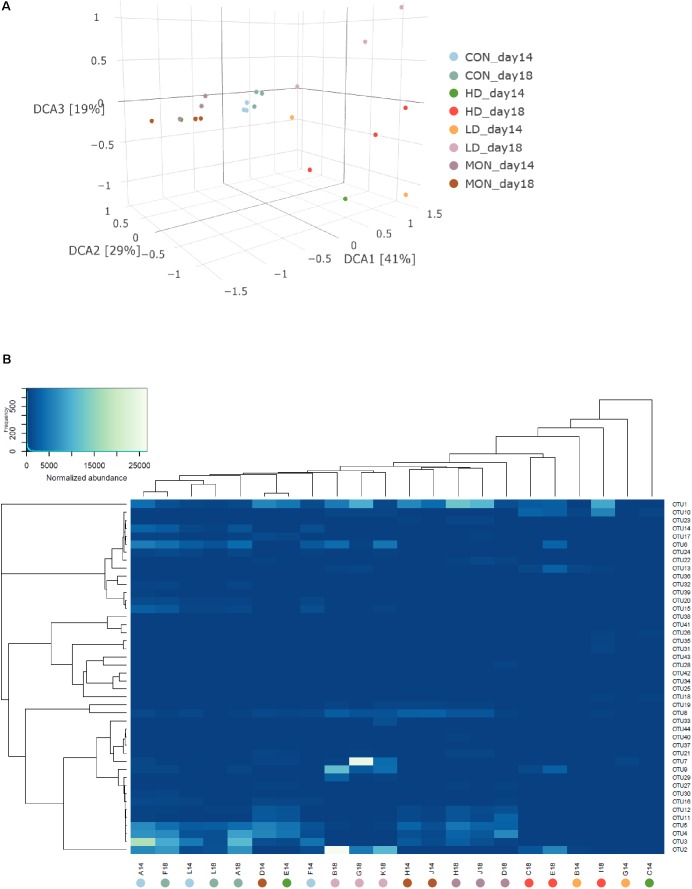
Analysis of microbial communities under different treatment conditions. **(A)** Detrended coordinate analysis of beta-diversity using unifrac distances. **(B)** Clustering of samples based on normalized abundances and phylogenetic predictions of OTUs. During the experimental period fermenters A, F, L received no addition (CON14, light blue), fermenters B, G, K were treated with 1 g of the experimental mixture (LD14, orange), fermenters C, E, I received 2 g of the experimental mixture (HD14, dark green) and 4.1 mg Monensin was added to fermenters D, J, H (MON14, dark purple). During the withdrawal period no additions were made (CON18, blue green; LD18, light purple; HD18, red; MON18, brown).

Due to the missing samples for LD14 and HD14, richness and Shannon diversity were only compared statistically for day 18 (**Figure 3**). In the HD group a high variability was visible in the number of OTUs and Chao 1 index at days 14 and 18, indicating a reduction in the richness in one of the samples. However, no statistically significant differences were detected for the number of observed OTUs and Chao 1 index among treatment groups at day 18. In contrast, Shannon index was reduced at day 18 in both groups treated with the experimental mixture before (*P* < 0.05 compared with CON) and was intermediate in the MON group. Based on the β-diversity analysis a clear clustering of the CON samples from the experimental and withdrawal period was observed (**Figure 4A**). Furthermore, all MON samples clustered close together. Samples treated with both doses of the experimental mixture were not clearly separated from each other and more widespread in the plot. One sample from the HD14 group was located close to the MON treated samples (E14). The clustering of the samples based on OTU abundance and predictions of the phylogenetic tree indicated a high similarity of most CON samples (**Figure 4B**). Only sample F14 was separated from the other control samples. The highest similarity of these samples was present to LD18 samples. Except D14, all MON treated samples also clustered together in the analysis. The remaining LD14 and HD18 samples (except E14) exhibited lower similarities with the other samples and each other, likewise to their widespread behavior in the plot.

#### Effects of the Treatments on the Archaeal Community Composition

Applying the different treatments induced changes within the community of *Archaea* based on family level (**Supplementary Table S5**). In control samples the most abundant family was *Methanobacteriaceae* (61 and 70%, days 14 and 18, respectively), followed by *Thermoplasmatales Incertae Sedis* (35 and 29%) and *Methanomicrobiaceae* (3.4 and 0.8%). In LD14 and HD14 samples, the percentage of *Methanobacteriaceae* (47% each) was nearly equal to the abundance of *Thermoplasmatales Incertae Sedis* (46 and 51%, LD and HD); the number of *Methanomicrobiaceae* remained low (6.2 and 1.6%). While LD18 was comparable to control samples, in HD18 the family *Thermoplasmatales Incertae Sedis* dominated with 72%. In contrast, the MON samples exhibited a relatively high percentage of *Methanomicrobiaceae* (16 and 6.1% at days 14 and 18, respectively). Bar plots including bacterial phyla and bacterial families are given in **Supplementary Figure S1**.

**FIGURE 5:**
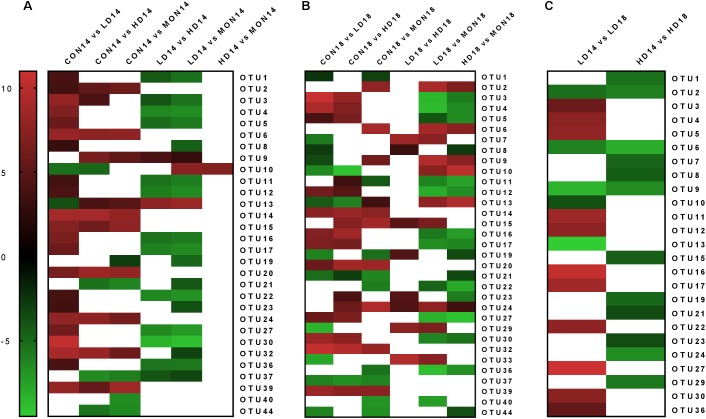
Significant changes (*P* < 0.05) in the normalized abundance of archaeal operational taxonomic units (OTUs). Differential OTU analysis based on normalized abundance counts contrasting the different experimental groups was performed using the software DESeq2 v1.12.4. *P*-values were corrected for multiple comparisons using Benjamini–Hochberg correction. In the heatmap, the log2 fold change of significantly different abundant OTUs (*P* < 0.05) is indicated by the red to green color scale. A threshold of ±2 was used for the log2 fold change. White areas indicate that no significant differences were observed, or the log2 fold change was between –2 and +2. Changes are depicted among treatment groups at day 14 (**A**, CON: control group, LD: 1 g of the experimental mixture per day, HD 2 g of the experimental mixture per day, MON: 4.1 mg Monensin per day), among treatment groups at day 18 (**B**, withdrawal period: no additions) and within treatment groups among days **(C)**.

Differential OTU analysis revealed differences (at least *P* < 0.05, log2 fold change ±2) for 30 archaeal OTUs at day 14 (**Figure 5A**). Eleven OTUs were statistically significantly reduced in fermenters treated with the low dose of the experimental mixture. Seven of these OTUs were closely related to genus *Methanobrevibacter* (OTUs 4, 5, 11, 12, 16, 27, 30) and 4 were assigned to *Thermoplasmatales Incertae Sedis*. Moreover, 7 OTUs were enriched in CON14 samples, OTU2, the most abundant OTU classified as genus *Methanobrevibacter*, and 6 OTUs from *Thermoplasmatales Incertae Sedis*. Surprisingly, only one OTU (OTU10) had a different abundance between the HD and MON samples. In general, OTU10 was more abundant in LD and HD samples compared to CON and MON samples. At day 18, 33 OTUs were significantly different in abundance among treatment groups (**Figure 5B**). There were still 4 OTUs enriched in CON group, 3 of them persistently enriched since day 14. Furthermore, 8 OTUs were reduced in LD and HD group compared to CON and MON treated samples, containing 6 of the 7 OTUs mentioned before classified as genus *Methanobrevibacter*, the closely related OTU3, as well as one OTU from *Thermoplasmatales Incertae Sedis*. Three *Methanobrevibacter*-OTUs were now enriched in LD samples and OTU 10 was still more abundant in LD and HD samples compared to CON and MON samples. Considering time-effect within groups, CON and MON group did not exhibit significant changes on OTU level between experimental and withdrawal period. Three OTUs showed an increase in the withdrawal period in LD and HD treatment, among them OTU2 and OTU6, which had also been enriched in CON14. Moreover, 9 OTUs increased in HD group and 2 increased in LD group, while 11 OTUs exhibited a decline the LD group (**Figure 5C**). There were only few statistically significant changes in bacterial OTUs (**Supplementary Table S6**).

#### Correlation of Archaeal OTU Abundance to Methane Production

To identify the most important OTUs responsible for the changes in methane production due to treatment, the normalized abundance of archaeal OTUs with an overall abundance of at least 1% was correlated to the daily production rate of methane. These OTUs were additionally compared to the 16S sequence data base of the JGI Integrated Microbial Genomes and Microbiomes (IMG/M) portal^2^ containing the sequences of the Hungate1000 collection (Seshadri et al., 2018). At day 14, 4 of 15 OTUs exhibited significant correlations with methane production. From genus *Methanobrevibacter*, significant positive correlations were detected for OTU3 (*P* < 0.001, *r* = 0.903) and OTU9 (*P* = 0.0058, *r* = 0.8182). These OTUs were closest matched to *Methanobrevibacter thaueri* DSM 11995 (99% sequence identity) and *Methanobrevibacter boviskoreani* JH1 (99% identity) by BLAST (**Table 2**), respectively. Moreover, OTU6 and OTU14 from the family *Thermoplasmatales Incertae Sedis* were positively correlated with methane production (*P* = 0.0202, *r* = 0.7333 and *P* = 0.0105, *r* = 0.7818, respectively). At day 18, only OTU5 (*Methanobrevibacter millerae* DSM 16643, 100% identity) was correlated to methane production (*P* = 0.0373, *r* = 0.6154).

**Table 2:** Best blast hits of the 15 most abundant operational taxonomic units (OTUs) using the JGI Integrated Microbial Genomes and Microbiomes (IMG/M) portal.

OTU	Identity	Gene ID	Best Blast hit
OTU1	94%	2667993422	Unclassified archaeon ISO4-G1
OTU2	99%	2553938470	*Methanobrevibacter boviskoreani* JH1
OTU3	99%	2622832649	*Methanobrevibacter thaueri* DSM 11995
OTU4	100%	2667995981	*Methanobrevibacter* sp. YE315
OTU5	100%	2595205797	*Methanobrevibacter millerae* DSM 16643
OTU6	99%	2667993422	Unclassified archaeon ISO4-G1
OTU7	99%	2682306483	*Methanobrevibacter olleyae* YLM1
OTU8	100%	2574179505	*Methanomicrobium mobile* DSM 1539
OTU9	98%	2553938470	*Methanobrevibacter boviskoreani* JH1
OTU10	99%	2667993422	Unclassified archaeon ISO4-G1
OTU11	100%	2622832649	*Methanobrevibacter thaueri* DSM 11995
OTU12	99%	2667995981	*Methanobrevibacter* sp. YE315
OTU13	95%	2637998119	Candidatus *Methanoplasma termitum* Mpt1
OTU14	99%	2667993422	Unclassified archaeon ISO4-G1
OTU15	94%	2637998119	Candidatus *Methanoplasma termitum* Mpt1

## Discussion

Although many compounds and dietary strategies have already been evaluated for their methane reducing potential, there is still a lack of practical solutions for livestock industry. Here, Mootral, a combination of a garlic powder and bitter orange extracts was tested for its effects on methane production, ruminal fermentation and the community composition of archaea.

### Mootral Effectively Reduced Methane Production Without Impairing Ruminal Fermentation

Garlic and several of its organosulphur compounds have been intensively studied in short-term *in vitro* culture or continuous flow experiments. These studies consistently indicated the methane reducing potential of garlic (Chaves et al., 2008; Patra and Yu, 2015), while the effects on SCFA were more variable. In the present experiment we observed an increased in the total SCFA production and in the molar proportion of butyrate and isovalerate. Previous studies, also observed enhanced total SCFA concentrations by moderate concentrations of garlic oil (Busquet et al., 2005a, 2006), moreover, most studies report an increase in the molar proportion of butyrate, often accompanied by a decreased proportion of acetate, while the effects on other SCFA and digestibilities may vary (Busquet et al., 2005a, 2006; Chaves et al., 2008; Patra and Yu, 2015). Variations in the concentrations and effects of the individual substances in the garlic extracts (Busquet et al., 2005b) and the diet type (Mateos et al., 2013) may contribute to the discrepancies. In sheep, allicin, one of the main active compounds in garlic, increases total SCFA concentration, OM and fiber digestibility, while decreasing the proportion of acetate and increasing the proportion of butyrate (Ma et al., 2016). Here, the proportion of acetate decreased only numerically, however, OM degradation was also enhanced fitting well with the elevated SCFA production. Several flavonoids have also been demonstrated to exhibit varying effects on rumen fermentation, besides their methane inhibiting potential (Oskoueian et al., 2013). A commercial bitter orange and grapefruit extract containing high amounts of naringin increases the total SCFA concentration in heifers while lowering the percentage of acetate and increasing the percentage of propionate whilst stabilizing the rumen against acidosis induction by enhancing growth of the lactate-utilizing *Megasphaera elsdenii* (Balcells et al., 2012). Patra and Yu (2015) already demonstrated that combining two or three compounds can enhance their methane-inhibiting potential, and this also appears to be the case for the tested combination of garlic and bitter orange compounds. The results of the present Rusitec trial as a long-term *in vitro* system clearly revealed a strong potential of the investigated mixture to reduce methane production without negatively effecting fermentation parameters such as SCFA production and degradation of OM. The increases in total SCFA production and in the molar percentage of butyrate observed in the present study are in line with the previous *in vitro* studies and *in vivo* studies on garlic compounds and the citrus extract. Based on the SCFA production this mixture appears to exhibit some beneficial effects on the bacterial community which were not focused on in this study, but might be of interest for further research. Hydrogen production estimated by calculation based on the SCFA concentration was not inhibited by the experimental compound like for monensin, instead it was enhanced. As ruminal fermentation was not impaired another hydrogen sink appears to be stimulated. The mechanism of methane reduction by Mootral does appear to not rely on a shift of the hydrogen production toward a propionate-producing bacterial community, but might rather be based on a direct effect on the ruminal archaeal community.

### Mootral Induced Changes in the Archaeal Community Composition

To investigate the changes of the archaeal community related to the experimental treatment SSCP analysis and subsequently Illumina MiSeq 16S rRNA amplicon sequencing were performed. The high similarity of CON14 and CON18 samples observed with both methods indicated a high stability of the archaeal community in the RUSITEC system along the timeline under control conditions. Furthermore, both methods revealed a shift of the archaeal community with the Mootral treatment, which was different to the shift observed with Monensin treatment. In the Illumina analysis, one HD14 sample (E14) clustered close to the MON14 group, as this effect was not observed for the SSCP analysis, this might be due to a contamination of this sample during sample preparation or transport for the sequencing and might also explain the low number of differentially abundant OTUs between these two groups. Results regarding HD14 might therefore be misleading and will not further be discussed. While Monensin changed the archaeal community composition during the whole experiment, the effect of Mootral appears to be more transient for the LD treatment, which was not different from control samples in the SSCP analysis at day 18, and nearest related to the control samples in the cluster analysis of the amplicon sequencing. For all Mootral treated samples from day 14 and the HD18 samples the sequencing indicated a higher variety than the SSCP analysis. Although garlic compounds have been intensively studied regarding methane production and biochemical properties, investigations addressing alterations of the archaeal community are rare. Quantitative evaluations report a suppressive effect of garlic and flavonoids on methanogen populations (Oskoueian et al., 2013; Patra and Yu, 2015; Ma et al., 2016). However, this effect has to be investigated by qPCR for the experimental compound. Regarding the community composition, garlic oil treated samples of rumen fluid exhibited a unique archaeal community composition in DGGE analysis (Patra and Yu, 2015). To our knowledge effects of citrus extract on the rumen archaeal community have not been investigated yet, however, other flavonoid-containing plant compounds may reduce archaeal richness and evenness (Oh et al., 2017) or affect the major groups of methanogens (Ramos-Morales et al., 2018). The high accordance of the results of both methods, which are also based on different primer pairs underline the reliability of the obtained results in the present study and the amplicon sequencing approach offers a detailed investigation on the effect of the experimental compound on the archaeal community.

### Several OTUs Can Be Linked to Specific Treatment Groups and Might Contribute to Differences in Methane Production

All archaea identified by the sequencing analysis belonged to the phylum *Euryarchaeota*. The family *Methanobacteria* with its genera *Methanobrevibacter* and *Methanosphaera* was dominant followed by *Thermoplasmatales, Incertae sedis*, and *Methanomicrobium*. These archaea are typically found in the rumen in *in vivo* and *in vitro* studies (Janssen and Kirs, 2008; Henderson et al., 2015; Duarte et al., 2017a,b). Several differences in the abundance of individual OTUs were detected among treatment groups. Differences in methane production might be related to the different properties for methane production of these OTUs and the respective archaeal groups. Uncultured *Thermoplasmatales* found in the rumen were formerly grouped under the name Rumen Cluster C (Janssen and Kirs, 2008), but recently have been proposed to represent a separate order of methylotrophic methanogenic archaea named *Methanoplasmatales* (Iino et al., 2013) or *Methanomassiliicoccaceae* (Paul et al., 2012). In the present study, most OTUs from *Thermoplasmatales* were closest related to Unclassified archeon ISO4-G1 and Canditatus *Methanoplasma termitum* Mpt1 in the Blast search. However, differences in the response to the treatments were observed among these OTUs. While two OTUs related to Unclassified archeon ISO4-G1 were correlated with methane production and more abundant in control samples, another OTU was enriched in the Mootral treated samples, suggesting that these OTUS are derived from different species which react differently to the treatment. These assumptions are in line with contradictory observations from previous investigations, one described a reduction of *Thermoplasmatales* being associated with lower methane emissions (Poulsen et al., 2013), while another study reported a higher abundance of *Thermoplasmatales* in cows emitting less methane (Danielsson et al., 2017). The abundance of *Thermoplasmatales*, or some members of *Thermoplasmatales* might also be negatively correlated to certain *Methanobrevibacter* spp. (Danielsson et al., 2017; McGovern et al., 2017). *Methanobrevibacter* are hydrogenotrophic methanogens (Leahy et al., 2013). However, differences in the occurrence of two isoforms for the methyl coenzyme M reductase (Mcr) enzyme have been detected among two major groups of rumen *Methanobrevibacter*. *Methanobrevibacter ruminantium* which forms together with *Methanobrevibacter olleyae* and relatives the RO clade only displays the Mcr I isoform which works at low H_2_; in contrast, members of the second group *Methanobrevibacter gottschalkii*, *Methanobrevibacter smithii*, *Methanobrevibacter millerae*, *Methanobrevibacter thaueri* (SGMT clade) additionally possess genes for the Mcr II isoform which is induced under high H_2_-pressure (McAllister et al., 2015; Tapio et al., 2017). Higher abundances of the SGMT clade have already been associated with higher methane emissions *in vivo* (Zhou et al., 2011; Danielsson et al., 2012; Danielsson et al., 2017), this agrees with the results of the present study were the abundances of OTUs closely matched to known strains of *Methanobrevibacter thaueri* and *Methanobrevibacter millerae* were correlated with methane production. The CON14 group exhibited an enrichment of an OTU related to *Methanobrevibacter boviskoreani* and this OTU and another related OTU increased in LD18 and HD18 samples. *Methanobrevibacter boviskoreani* is also a hydrogenotrophic methanogen which is able to replace *Methanobrevibacter smithii* in weaning pigs (Lee et al., 2013; Federici et al., 2015). In contrast, Mootral reduced the abundance of several OTUs from genus *Methanobrevibacter* in the LD14, LD18, and HD18 treatment. All of these sequences clustered closely to sequences from the SGMT clade in the IMG blast search, indicating that Mootral potentially reduced methane production by suppressing methanogen species of the SGMT clade.

## Conclusion

In the present study we tested a mixture of garlic powder and bitter orange extracts, Mootral, for its impact on rumen fermentation, methane production and the archaeal community by using the Rusitec. The mixture was effective in reducing methane production without impairing rumen fermentation. The SSCP analysis and amplicon sequencing data indicate an alteration of the archaeal community by the compound, particularly a suppression of the high methane-producing *Methanobrevibacter* spp. of the SGMT clade.

## Author Contributions

GB and MG designed the experiments. ME and SR data acquisition and statistical analysis. ME, SR, and MG wrote the paper. ME, SR, MG, and GB review and approved the final draft.

## Conflict of Interest Statement

MG was an employee of Neem Biotech Ltd. during the period in which the research was performed. The remaining authors declare that the research was conducted in the absence of any commercial or financial relationships that could be construed as a potential conflict of interest.

## Footnotes

1 https://img.jgi.doe.gov

2 https://img.jgi.doe.gov

## Supplementary Material

FIGURE S1 Percentage contribution of sequences (%) at phylum level **(A)** and at family level **(B)** to the total number of sequences. Data are presented as means.

TABLE S1 Crude nutrients of the substrates.

TABLE S2 Buffer composition.

TABLE S3 Archaeal operational taxonomic units (OTUs) sorted according to their overall relative abundance (Rel. Ab.).

TABLE S4 Bacterial operational taxonomic units (OTUs) sorted according to their overall relative abundance (Rel. Ab.).

TABLE S5 Percentage contribution of sequences (%) at domain level to the total number of sequences and on family level to the total number of archaeal sequences. During the experimental period (day 14) control fermenters (CON) received no addition, low dose group (LD) was treated with 1 g, high dose fermenters (HD) received 2 g of the experimental mixture and monensin was used as positive control (MON). During withdrawal period (day 18) no additions were made. Data are presented as means. Relative contribution of sequences was compared using One-way ANOVA followed by Tukey post-test for day 18 (day 14 was not compared due to missing samples). Significant differences among treatment groups in post-test are indicated by different superscripts.

TABLE S6 Significant log 2 fold changes (*P* < 0.05, log 2 fold change ± 2) of bacterial operational taxonomic units (OTUs).

## Abbreviations

CON: control group
EP: experimental phase
HD: high dose Mootral group
LD: low dose Mootral group
MON: Monensin group
NMDS: non-metric dimensional scaling
OM: organic matter
OTU: operational taxonomic unit
Rusitec: rumen simulation technique
SCFA: short chain fatty acid
SSCP: single strand conformation polymorphism
WP: withdrawal phase.
