# Supplementary material for: Application of MootralTM Reduces Methane Production by Altering the Archaea Community in the Rumen Simulation Technique
Source: Front Microbiol. 2018 Sep 4;9:2094. doi: 10.3389/fmicb.2018.02094 (PMC6132076; doi:10.3389/fmicb.2018.02094)
Supplement: FIGURE S1 — Percentage contribution of sequences (%) at phylum level (A) and at family level (B) to the total number of sequences. Data are presented as means. [file Data_Sheet_1.PDF]

Supplementary Material

Application of Mootral™ reduces methane production by altering the Archaea community in the rumen simulation technique

Melanie Eger*, Michael Graz, Susanne Riede, Gerhard Breves

*** Correspondence:** Corresponding Author: Melanie.Eger@tiho-hannover.de

1. Supplementary Figures

Supplementary Figure S1

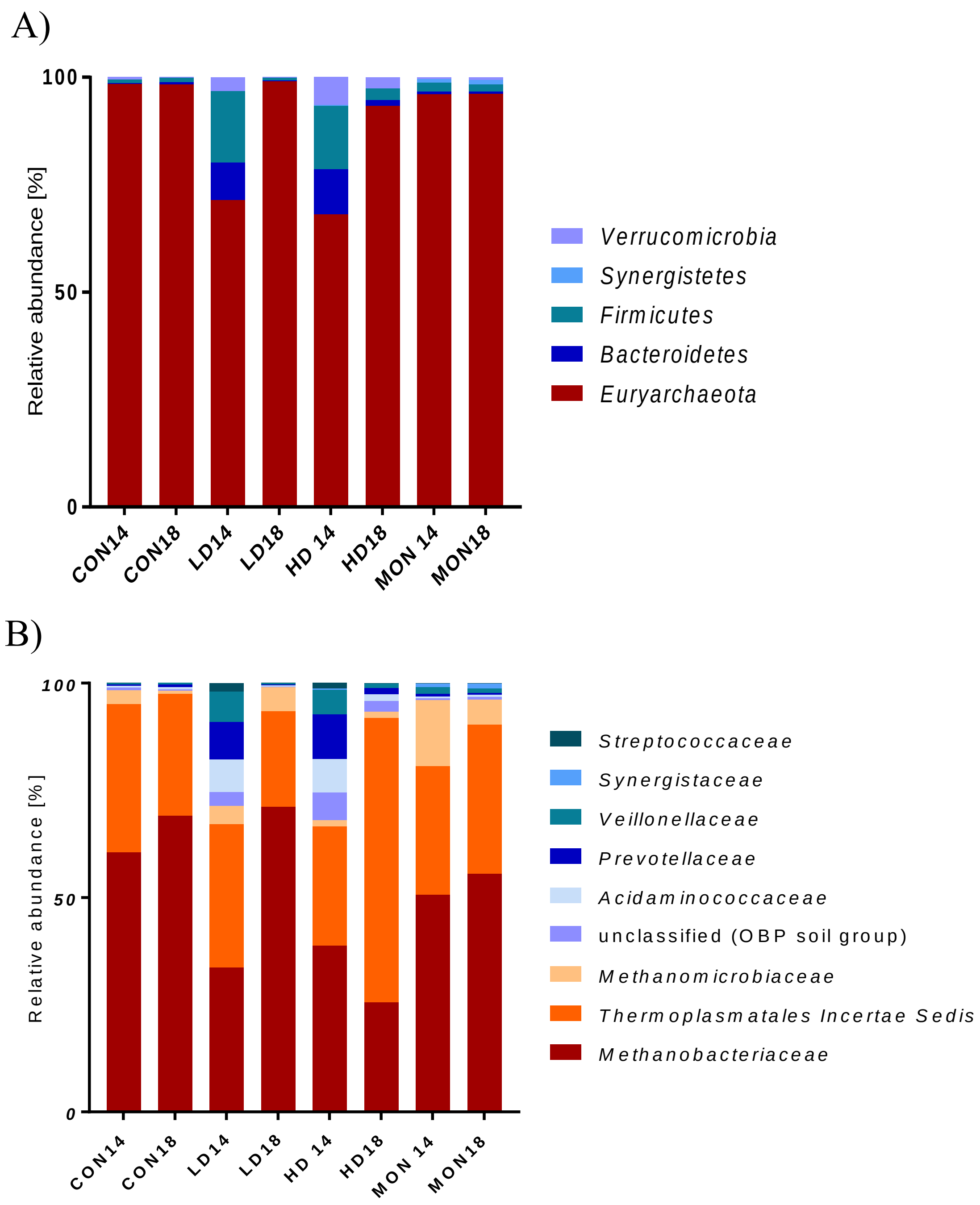

Supplementary Figure 1. Percentage contribution of sequences (%) at phylum level (A) and at family level (B) to the total number of sequences. Data are presented as means.
