# Supplementary material for: Application of MootralTM Reduces Methane Production by Altering the Archaea Community in the Rumen Simulation Technique
Source: Front Microbiol. 2018 Sep 4;9:2094. doi: 10.3389/fmicb.2018.02094 (PMC6132076; doi:10.3389/fmicb.2018.02094)
Supplement: TABLE S1 — Crude nutrients of the substrates. [file Table_1.docx]

Supplementary Material

Application of Mootral™ reduces methane production by altering the Archaea community in the rumen simulation technique

**Melanie Eger*, Michael Graz, Susanne Riede, Gerhard Breves**

*** Correspondence:** Corresponding Author: [Melanie.Eger@tiho-hannover.de](mailto:Melanie.Eger@tiho-hannover.de)

**2. Supplementary Tables**

Supplementary Table S1: Crude nutrients of the substrates.

|  | Hay | Concentrate | Experimental mixture |
| --- | --- | --- | --- |
| Dry matter [%] | 91.2 | 90.5 | 95.1 |
| Crude ash [%] | 6.5 | 6.7 | 3.3 |
| Crude protein [%] | 9.6 | 17.9 | 24.0 |
| Crude fat [%] | 1.0 | 3.7 | 0.6 |
| Crude fiber [%] | 28.8 | 7.2 | 0 |
