# Supplementary material for: Application of MootralTM Reduces Methane Production by Altering the Archaea Community in the Rumen Simulation Technique
Source: Front Microbiol. 2018 Sep 4;9:2094. doi: 10.3389/fmicb.2018.02094 (PMC6132076; doi:10.3389/fmicb.2018.02094)
Supplement: TABLE S2 — Buffer composition. [file Table_2.docx]

Supplementary Material

Application of Mootral™ reduces methane production by altering the Archaea community in the rumen simulation technique

**Melanie Eger*, Michael Graz, Susanne Riede, Gerhard Breves**

*** Correspondence:** Corresponding Author: [Melanie.Eger@tiho-hannover.de](mailto:Melanie.Eger@tiho-hannover.de)

Supplementary Table S2: Buffer composition

| Ingredient | Concentration [mM] |
| --- | --- |
| NaCl | 28.00 |
| KCl | 7.69 |
| HCl (1 N) | 0.50 |
| Ca_2_Cl ∙ 2 H_2_O | 0.22 |
| MgCl_2_ ∙ 6 H_2_O | 0.63 |
| NH_4_Cl | 5.00 |
| Na_2_HPO_4_ ∙ 12 H_2_O | 10.00 |
| NaH_2_PO_4_ ∙ H_2_O | 10.00 |
| NaHCO_3_ | 97.90 |
