# Supplementary material for: Application of MootralTM Reduces Methane Production by Altering the Archaea Community in the Rumen Simulation Technique
Source: Front Microbiol. 2018 Sep 4;9:2094. doi: 10.3389/fmicb.2018.02094 (PMC6132076; doi:10.3389/fmicb.2018.02094)
Supplement: TABLE S3 — Archaeal operational taxonomic units (OTUs) sorted according to their overall relative abundance (Rel. Ab.). [file Table_3.docx]

Supplementary Material

Application of Mootral™ reduces methane production by altering the Archaea community in the rumen simulation technique

**Melanie Eger*, Michael Graz, Susanne Riede, Gerhard Breves**

*** Correspondence:** Corresponding Author: [Melanie.Eger@tiho-hannover.de](mailto:Melanie.Eger@tiho-hannover.de)

Supplementary Table S3. Archaeal operational taxonomic units (OTUs) sorted according to their overall relative abundance (Rel. Ab.).

| OTU ID | Rel. Ab. [%] | Read length | Confidence | SILVA top hit (lowest taxonomic level based on at least 70% confidence) |
| --- | --- | --- | --- | --- |
| OTU1 | 17.4 | 379 | 100% | *Thermoplasmatales Incertae Sedis* (family) |
| OTU2 | 11.7 | 380 | 100% | *Methanobrevibacter* (genus) |
| OTU3 | 9.42 | 380 | 100% | *Methanobrevibacter* (genus) |
| OTU4 | 9.31 | 380 | 100% | *Methanobrevibacter* (genus) |
| OTU5 | 8.59 | 380 | 100% | *Methanobrevibacter* (genus) |
| OTU6 | 5.04 | 379 | 100% | *Thermoplasmatales Incertae Sedis* (family) |
| OTU8 | 4.95 | 376 | 100% | *Methanomicrobium* (genus) |
| OTU7 | 4.73 | 380 | 100% | *Methanobrevibacter* (genus) |
| OTU9 | 3.80 | 380 | 100% | *Methanobrevibacter* (genus) |
| OTU10 | 3.74 | 379 | 100% | *Thermoplasmatales Incertae Sedis* (family) |
| OTU11 | 2.76 | 380 | 100% | *Methanobrevibacter* (genus) |
| OTU12 | 2.31 | 380 | 100% | *Methanobrevibacter* (genus) |
| OTU13 | 1.47 | 379 | 100% | *Thermoplasmatales Incertae Sedis* (family) |
| OTU14 | 1.44 | 379 | 100% | *Thermoplasmatales Incertae Sedis* (family) |
| OTU15 | 1.37 | 379 | 100% | *Thermoplasmatales Incertae Sedis* (family) |
| OTU16 | 0.899 | 380 | 100% | *Methanobrevibacter* (genus) |
| OTU19 | 0.867 | 376 | 100% | *Methanomicrobium* (genus) |
| OTU17 | 0.865 | 379 | 100% | *Thermoplasmatales Incertae Sedis* (family) |
| OTU21 | 0.66 | 380 | 100% | *Methanosphaera* (genus) |
| OTU20 | 0.654 | 379 | 100% | *Thermoplasmatales Incertae Sedis* (family) |
| OTU24 | 0.638 | 379 | 100% | *Thermoplasmatales Incertae Sedis* (family) |
| OTU23 | 0.607 | 379 | 100% | *Thermoplasmatales Incertae Sedis* (family) |
| OTU22 | 0.606 | 379 | 100% | *Thermoplasmatales Incertae Sedis* (family) |
| OTU27 | 0.452 | 380 | 100% | *Methanobrevibacter* (genus) |
| OTU29 | 0.41 | 380 | 100% | *Methanobrevibacter* (genus) |
| OTU30 | 0.41 | 380 | 100% | *Methanobrevibacter* (genus) |
| OTU32 | 0.345 | 382 | 100% | *Thermoplasmatales Incertae Sedis* (family) |
| OTU33 | 0.271 | 380 | 99% | Methanogenic archeon LGM-SL1 (species) |
| OTU36 | 0.185 | 382 | 100% | *Thermoplasmatales Incertae Sedis* (family) |
| OTU37 | 0.183 | 380 | 100% | *Methanosphaera* (genus) |
| OTU40 | 0.141 | 380 | 100% | *Methanosphaera* (genus) |
| OTU39 | 0.139 | 379 | 100% | *Thermoplasmatales Incertae Sedis* (family) |
| OTU44 | 0.13 | 380 | 100% | *Methanosphaera* sp. ISO3-F5 (species) |
