# Supplementary material for: Application of MootralTM Reduces Methane Production by Altering the Archaea Community in the Rumen Simulation Technique
Source: Front Microbiol. 2018 Sep 4;9:2094. doi: 10.3389/fmicb.2018.02094 (PMC6132076; doi:10.3389/fmicb.2018.02094)
Supplement: TABLE S4 — Bacterial operational taxonomic units (OTUs) sorted according to their overall relative abundance (Rel. Ab.). [file Table_4.DOCX]

Supplementary Material

Application of Mootral™ reduces methane production by altering the Archaea community in the rumen simulation technique

**Melanie Eger*, Michael Graz, Susanne Riede, Gerhard Breves**

*** Correspondence:** Corresponding Author: [Melanie.Eger@tiho-hannover.de](mailto:Melanie.Eger@tiho-hannover.de)

Supplementary Table S4. Bacterial operational taxonomic units (OTUs) sorted according to their overall relative abundance (Rel. Ab.).

| OTU_  ID | Rel. Ab. [%] | Phylum | Class | Order | Family | Genus | Species |
| --- | --- | --- | --- | --- | --- | --- | --- |
| OTU18 | 0.72 | *Firmicutes* | *Negativicutes* | *Selenomonadales* | *Acidaminococcaceae* | *Succiniclasticum* |  |
| OTU31 | 0.539 | *Verruco-microbia* | OPB35 soil  group |  |  |  |  |
| OTU25 | 0.5 | *Firmicutes* | *Negativicutes* | *Selenomonadales* | *Veillonellaceae* | *Megasphaera* | *Megasphaera* sp. BS-4 |
| OTU26 | 0.43 | *Bacteroidetes* | *Bacteroidia* | *Bacteroidales* | *Prevotellaceae* | *Prevotella* 1 | Unidentified rumen  Bacterium RFN21 |
| OTU28 | 0.375 | *Synergistetes* | *Synergistia* | *Synergistales* | *Synergistaceae* | *Synergistes* |  |
| OTU34 | 0.263 | *Firmicutes* | *Negativicutes* | *Selenomonadales* | *Veillonellaceae* | *Schwartzia* | *Schwartzia succinivorans* |
| OTU35 | 0.229 | *Verruco-microbia* | OPB35 soil group |  |  |  |  |
| OTU38 | 0.163 | *Bacteroidetes* | *Bacteroidia* | *Bacteroidales* | *Prevotellaceae* | *Prevotella* 1 |  |
| OTU41 | 0.124 | *Bacteroidetes* | *Bacteroidia* | *Bacteroidales* | *Prevotellaceae* | *Prevotella* 1 |  |
| OTU42 | 0.111 | *Firmicutes* | *Negativicutes* | *Selenomonadales* | *Veillonellaceae* | *Anaerovibrio* | Unidentified rumen  Bacterium RFN69 |
| OTU43 | 0.110 | *Firmicutes* | *Bacilli* | *Lactobacillales* | *Streptococcaceae* | *Streptococcus* | unidentified |
