# Supplementary material for: Application of MootralTM Reduces Methane Production by Altering the Archaea Community in the Rumen Simulation Technique
Source: Front Microbiol. 2018 Sep 4;9:2094. doi: 10.3389/fmicb.2018.02094 (PMC6132076; doi:10.3389/fmicb.2018.02094)
Supplement: TABLE S5 — Percentage contribution of sequences (%) at domain level to the total number of sequences and on family level to the total number of archaeal sequences. During the experimental period (day 14) control fermenters (CON) received no addition, low dose group (LD) was treated with 1 g, high dose fermenters (HD) received 2 g of the experimental mixture and monensin was used as positive control (MON). During withdrawal period (day 18) no additions were made. Data are presented as means. Relative contribution of sequences was compared using One-way ANOVA followed by Tukey post-test for day 18 (day 14 was not compared due to missing samples). Significant differences among treatment groups in post-test are indicated by different superscripts. [file Table_5.DOCX]

Supplementary Material

Application of Mootral™ reduces methane production by altering the Archaea community in the rumen simulation technique

**Melanie Eger*, Michael Graz, Susanne Riede, Gerhard Breves**

*** Correspondence:** Corresponding Author: [Melanie.Eger@tiho-hannover.de](mailto:Melanie.Eger@tiho-hannover.de)

Supplementary table 5. Percentage contribution of sequences (%) at domain level to the total number of sequences and on family level to the total number of archaeal sequences. During the experimental period (day14) control fermenters (CON) received no addition, low dose group (LD) was treated with 1 g, high dose fermenters (HD) received 2 g of the experimental mixture and monensin was used as positive control (MON). During withdrawal period (day 18) no additions were made. Data are presented as means. Relative contribution of sequences was compared using One-way ANOVA followed by Tukey post-test for day 18 (day 14 was not compared due to missing samples). Significant differences among treatment groups in post-test are indicated by different superscripts.

| Taxonomic level | day | Treatment | | | | pooled  SD | *P* value |
| --- | --- | --- | --- | --- | --- | --- | --- |
|  |  | CON | LD | HD | MON |  |  |
| **Kingdom [% of total sequences]** | | | | | | | |
| Archaea | 14 | 98.41 | 71.42 | 68.09 | 96.03 | 20.3 | n.d.^1^ |
|  | 18 | 98.28^a^ | 99.03^a^ | 93.28^a^ | 96.2^a^ | 2.54 | 0.090 |
| Bacteria | 14 | 1.60 | 28.54 | 31.9 | 3.94 | 20.3 | n.d. |
|  | 18 | 1.76^a^ | 1.00^a^ | 6.69^a^ | 3.79^a^ | 2.55 | 0.096 |
| **Family [% of archaeal sequences]** | | | | | | | |
| Methanobacteriaceae | 14 | 61.49 | 47.43 | 47.17 | 52.62 | 23.5 | n.d. |
|  | 18 | 70.26^a^ | 71.88^a^ | 26.75^b^ | 58.01^ab^ | 16.4 | 0.033 |
| Methanomicrobiaceae | 14 | 3.401 | 6.18 | 1.57 | 16.09 | 6.62 | n.d. |
|  | 18 | 0.82^a^ | 5.71^a^ | 1.56^a^ | 6.11^a^ | 2.08 | 0.027 |
| Thermoplasmatales Incertae Sedis | 14 | 35.1 | 46.39 | 51.26 | 31.29 | 24.7 | n.d. |
|  | 18 | 28.93^a^ | 22.41^a^ | 71.69^b^ | 35.88^ab^ | 14.9 | 0.015 |

^1)^ not determined
