# Supplementary material for: Application of MootralTM Reduces Methane Production by Altering the Archaea Community in the Rumen Simulation Technique
Source: Front Microbiol. 2018 Sep 4;9:2094. doi: 10.3389/fmicb.2018.02094 (PMC6132076; doi:10.3389/fmicb.2018.02094)
Supplement: TABLE S6 — Significant log 2 fold changes (P < 0.05, log 2 fold change ± 2) of bacterial operational taxonomic units (OTUs). [file Table_6.DOCX]

Supplementary Material

Application of Mootral™ reduces methane production by altering the Archaea community in the rumen simulation technique

**Melanie Eger*, Michael Graz, Susanne Riede, Gerhard Breves**

*** Correspondence:** Corresponding Author: [Melanie.Eger@tiho-hannover.de](mailto:Melanie.Eger@tiho-hannover.de)

Supplementary Table S6. Significant log 2 fold changes (*P* < 0.05, log 2 fold change ± 2) of bacterial operational taxonomic units (OTUs).

| Day 14 | CON vs. LD | CON vs HD | CON vs MON | LD vs HD | LD vs MON | HD vs MON |
| --- | --- | --- | --- | --- | --- | --- |
| OTU25 |  | -2.7 | -3.3 |  |  |  |
| OTU26 | -2.0 | -3.0 |  |  |  |  |
| OTU28 |  | -7.1 | -8.5 | -5.0 | -6.4 |  |
| OTU38 |  |  |  |  | 2.3 |  |
| OTU43 | -2.9 | -2.4 |  |  |  |  |
| Day 18 | | | | | | |
| OTU25 |  |  | -3.3 |  | -2.7 |  |
| OTU28 |  |  | -8.2 |  | -8.8 | -6.3 |
| OTU31 |  | -2.5 |  |  |  |  |
| OTU35 |  | -3.9 |  | -3.1 |  | 2.9 |
| OTU38 |  |  |  |  | 2.1 | 2.0 |
| OTU42 |  | -3.1 |  |  |  |  |
